# Assessment of the temporal trend and daily profiles of the dietary purine intake among Chinese residents during 2014 to 2021

**DOI:** 10.3389/fnut.2023.1259053

**Published:** 2023-11-09

**Authors:** Shiwen Li, Xin Liu, Xiwu Jia, Min Fang, Qing Yang, Zhiyong Gong

**Affiliations:** College of Food Science and Engineering, Key Laboratory for Deep Processing of Major Grain and Oil (The Chinese Ministry of Education), Hubei Key Laboratory for Processing and Transformation of Agricultural Products, Wuhan Polytechnic University, Wuhan, China

**Keywords:** purine, dietary intake, Chinese residents, temporal trend, joinpoint analyzes

## Abstract

The incidence of hyperuricemia is on the rise in China, primarily due to dietary habits. However, limited data exists regarding dietary purine intake in the country. This study aimed to estimate the daily dietary purine intake among Chinese residents from 2014 to 2021 and evaluate the temporal trend using joinpoint regression analysis. The analysis revealed an annual percentage change (APC) of 0.8% (95% CI: 0.1–1.5%) in dietary purine intake prior to the joinpoint (2014–2019). Following the joinpoint (2019–2021), the APC significantly increased to 6.5% (95% CI: 3.3–9.8%), indicating a noteworthy upward trend (*p* = 0.045). Furthermore, the average daily purine intake varied significantly among different regions of China, with the southern region showing the highest dietary intake of purines. Considering the diverse contributions of various food sources to dietary purine intake, it was observed that meat consumption had the greatest impact, accounting for 36.2% of purine intake, followed by cereals consumption (25.3%) and vegetables and edible fungi (24.2%). These findings hold significance for dietary intervention and management strategies aimed at reducing purine intake among the population.

## 1. Introduction

Purine, an essential base, plays a crucial role as a building block of deoxyribonucleic acid (DNA) and ribonucleic acid (RNA) in living organisms (1). It consists primarily of guanine, adenine, hypoxanthine, and xanthine, which are commonly found in various food sources. However, excessive dietary intake of purines can lead to elevated serum uric acid levels and pose a significant risk of developing hyperuricemia. In the human body, purines are eventually oxidized into uric acid (2, 3). Roughly 20% of the uric acid in the body is derived from purine-rich foods, while the remaining 80% originates from the synthesis of amino acids, nucleotides, other small-molecule compounds, and the catabolism of nucleic acids (4). Lack of timely excretion of uric acid can lead to its deposition in human joints and other tissues, causing hyperuricemia, gout, and other associated diseases (5–7).

Epidemiological studies have particularly focused on dietary interventions as important tools to prevent or slow down the adverse prognosis of gout (8). With economic development and changes in dietary habits, the prevalence of hyperuricemia and gout is increasing yearly and affecting the younger population (9). Hyperuricemia has become the “fourth highest” after diabetes, hypertension, and hyperlipidemia (10). In 2021, a white paper on high uric acid and gout trends in China revealed an overall prevalence of hyperuricemia of 13.3%, affecting approximately 177 million people, and an overall incidence of gout of 1.1%, affecting approximately 14.66 million people. Other data show that nearly 60% of patients with hyperuricemia and gout in China are aged 18–35.^1^ Thus, gout is no longer a disease affecting the middle-aged and old. The young population is suggested to incorporate the prevention and treatment of gout into their knowledge, especially those who prefer eating high-purine foods such as seafood, drinking excessively, working and resting irregularly, being overweight, and smoking (11).

For a long time, dietary purine content has been debated as the leading cause of hyperuricemia and gout in the academic community (12). However, most studies suggest that a high-purine diet increases the serum uric acid content, which is implicated in hyperuricemia and gout (13–15). In addition to drug therapy, a low-purine diet is key to treating patients with gout. Further research on hyperuricemia and gout, as well as the purine content in foods, is imperative. It has been reported that purine intake in patients with acute and chronic gout should be <150 mg/day (16); thus, it is important to know the daily intake of dietary purine. However, few reports evaluate purine intake in Chinese residents’ diet.

Socioeconomic development has led to significant improvements in living standards and changes in the dietary structure of Chinese residents. Urbanization and agricultural policies have played a role in shaping changes in the dietary structure of Chinese residents, which may greatly influence the dietary intake of purine and the management of purine-related chronic diseases (17). Therefore, it is essential to analyze the temporal trends and differences in purine intake in Chinese residents.

This study evaluated the national level of total purine intake from commonly consumed foods of Chinese residents. Based on the statistics of different kinds of food consumption from 2014 to 2021 and the purine content database, a temporal trend was also assessed for the purine intake from different foods.

## 2. Materials and methods

### 2.1. Data source for food consumption of the Chinese population

National-level dietary information of Chinese residents from 2014 to 2021 was obtained from the China Statistical Yearbooks, published by the National Bureau of Statistics. Specific variables were meticulously chosen to represent various dietary components and trends pertinent to the study objectives. The national statistical data included 10 categories of staple foods: grain, oil, vegetables and edible fungi, livestock meat, poultry, aquatic products, eggs, dairy products, fruits, and sugar. This data has been updated to the year 2021. A detailed breakdown of this dataset, including the presentation format, variables, and categories, can be found in Supplementary Tables S1–S3. We utilized a comprehensive dataset from this database, which offers detailed information on dietary patterns from 2014 to 2021. The staple food consumption data in the China Statistical Yearbooks covered 31 provincial regions stratified by area (urban/rural) and gender. Various sampling methods were comprehensively applied to the sample size of 66,000 urban and 74,000 rural households to investigate their main food consumption (18). All the data reported in this study were analyzed in adherence to relevant guidelines and regulations of National Bureau of Statistics of China.

### 2.2. Purine content in different food

Food purine contents were obtained from the Chinese food composition tables (19). Purines, including adenine, hypoxanthine, guanine, and xanthine in different foods, have been measured using high-performance liquid chromatography (HPLC) (20). Total purine content was the sum of adenine, guanine, hypoxanthine, and xanthine levels. The approximate values of specific purine content in each food are shown in Supplementary Table S1. For each type of food, several foods frequently consumed by residents in various categories were selected. The average purine content of each type of food was calculated (Supplementary Table S2).

### 2.3. Estimated dietary intake of purine

Foods frequently consumed by Chinese residents in each food category were selected, and the average purine content was calculated. Based on the amount of food consumed and the purine content of the food in each category, the estimated dietary intake (EDI) of purine in food is calculated as follows (21):

EDI=C×IR

EDI, estimated daily intake (mg/d); C, purine concentration in foods (mg/100 g); IR, intake rate (g/d).

### 2.4. Statistical analysis

Temporal trends of dietary purine intake were analyzed using joinpoint regression analysis, a statistical method that fits a series of joined straight lines between statistically significant changes in trend (joinpoints) and estimates the change between joinpoints (22). The analysis was performed using National Cancer Institute (NCI) Joinpoint Regression Program software (Version 4.1.1). Areas covered by population-based purine intake were classified into urban/rural areas and 31 administrative regions of the Chinese mainland, according to the National Bureau of Statistics of China. Urban areas were defined as administrative divisions encompassing larger territorial and population scales, including municipalities, prefecture-level cities. In contrast, rural areas referred to lower-tier administrative units such as counties, county-level cities, and districts. Data are expressed as mean ± standard deviation (SD). Statistical analysis was performed using Origin Lab software (version 2020). Geographic spatial data were visualized using ArcGIS software (version 10.4).

## 3. Results

### 3.1. Trends in daily dietary intake of purine for Chinese residents from 2014 to 2021

**Figure 1:**
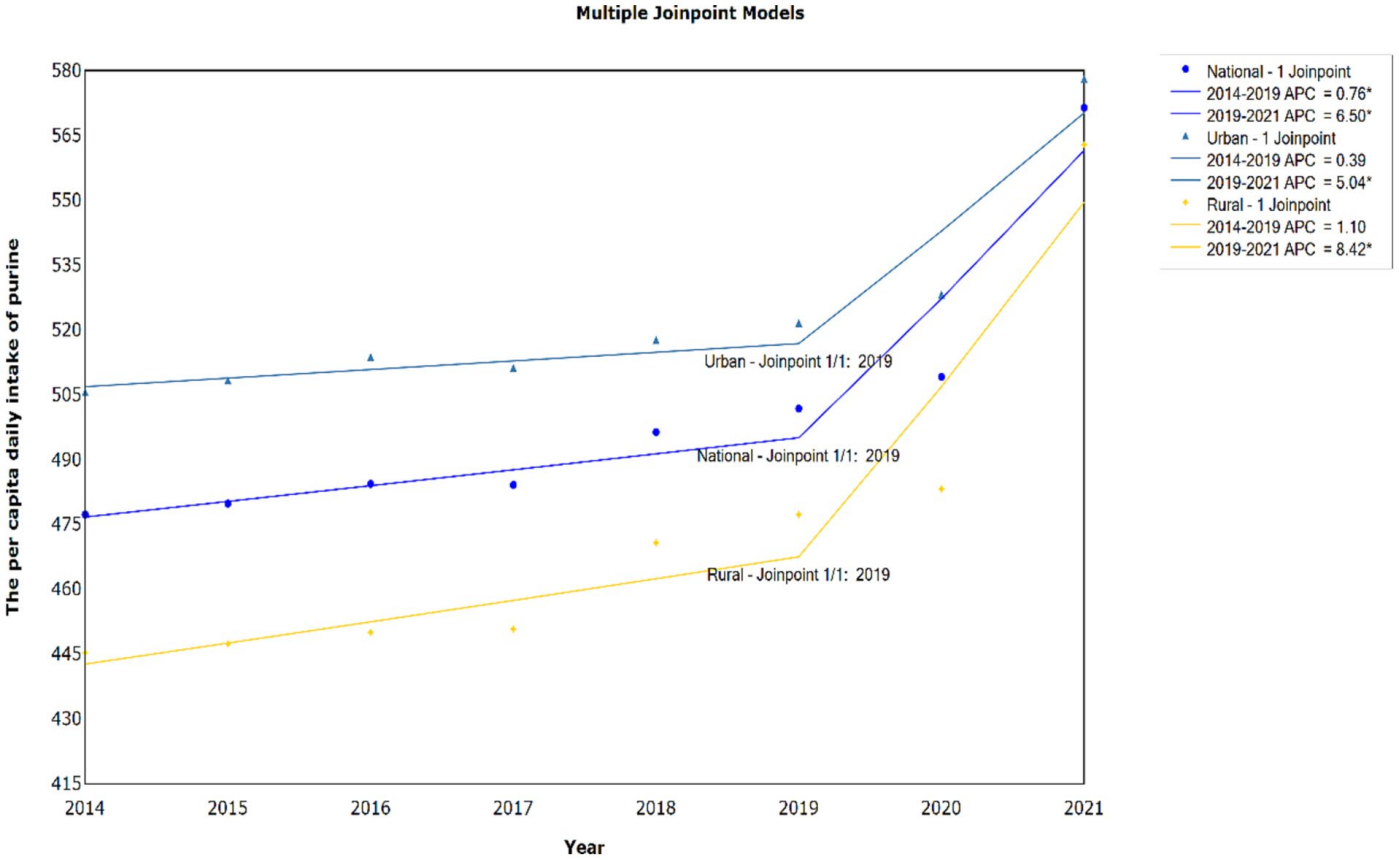
Joinpoint trends for *per capita* daily purine intake in Chinese residents, 2014–2021.

We conducted a joinpoint regression analysis to examine the dietary purine intake trend from 2014 to 2021 (as shown in Figure 1). The analysis identified one significant joinpoint in the year 2019. The residents in urban areas have shown higher *per capita* daily purine intake than that in rural areas. It was observed that the difference in *per capita* daily purine intake between urban and rural areas is gradually narrowing from 2014 to 2021. The joinpoint regression parameters are displayed in Table 1. At the national level, the annual percentage change (APC) in dietary purine intake was 0.8% (95% CI: 0.1–1.5%) before the joinpoint (2014–2019), and the APC dramatically increased to 6.5% (95% CI: 3.3–9.8%) after the joinpoint (2019–2021), indicating a significant increasing trend (*p* = 0.045). The annual average percent change (AAPC) for the entire study period (2014–2021) was 2.4% (95% CI: 1.7–3.0%). As for the trend at urban level, the APC in dietary purine intake was 0.4% (95% CI: −0.0–0.8%). After the joinpoint (2019–2021), the APC increased to 5.0% (95% CI: 3.0–7.1%), indicating a significant increasing trend (*p* = 0.038). A non-significant increasing trend was observed for dietary purine intake in rural area. While the AAPC of the rural level is greater than urban level (3.1% vs. 1.7%).

**Table 1:** Joinpoint analysis of *per capita* daily purine intake in urban, rural, and national areas.

	Total study period		Period 1			Period 2			*p*-value
	AAPC (%)	95% CI	Years	APC (%)	95% CI	Years	APC (%)	95% CI	
National	2.4	1.7–3.0	2014–2019	0.8	0.1–1.5	2019–2021	6.5	3.3–9.8	0.045
Urban	1.7	1.3–2.1	2014–2019	0.4	−0.0-0.8	2019–2021	5.0	3.0–7.1	0.038
Rural	3.1	1.8–4.4	2014–2019	1.1	−0.3-1.8	2019–2021	8.4	2.5–15.4	0.075

AAPC, annual average percent change; APC, annual percent change; CI, annual percent change.

### 3.2. Trends of dietary intake of purine by different foods from 2014 to 2021

**Figure 2:**
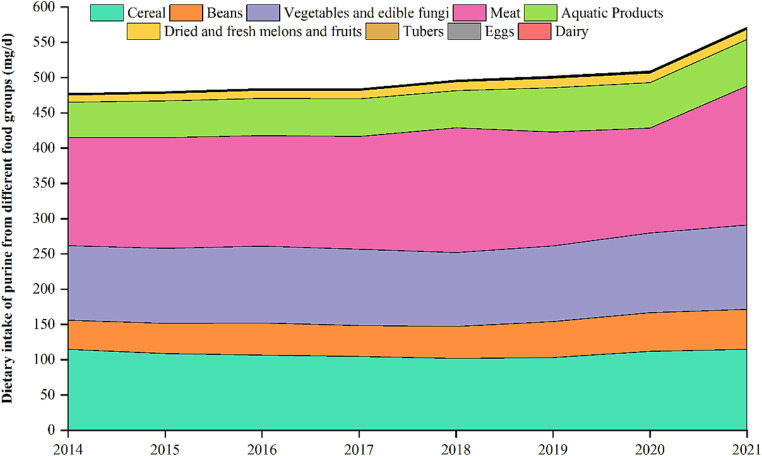
Trends in dietary purine intake from different foods, 2014–2021.

Figure 2 shows trends in dietary purine intake in different food groups from 2014 to 2021. Dietary purine intake from cereals had a low level of 101.6 mg in 2018 and a high level of 114.8 mg in 2014. Purine intake from vegetables and edible fungi increased from 2014 to 2021, reaching a peak of 119.6 mg in 2021. Purine intake from Meat gradually increased, reaching a peak of 197.2 mg in 2021. Purine intake from aquatic products showed an overall increasing trend, peaking at 65.8 mg in 2021. Purine intake from beans increased, reaching a peak of 56.6 mg in 2021.

### 3.3. The averaged geographical distribution of dietary purine intake from 2014 to 2021

**Figure 3:**
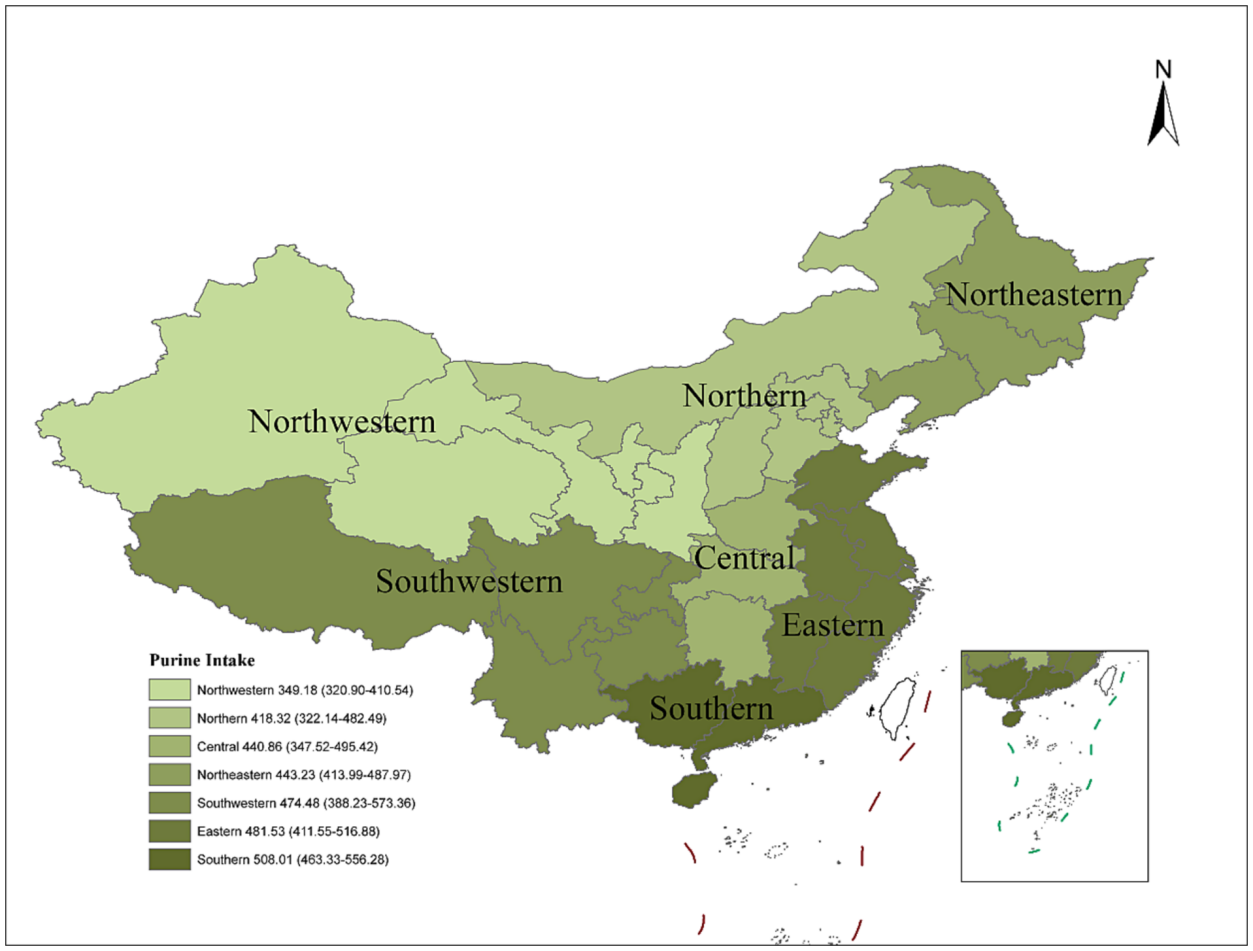
The averaged geographical distribution of dietary purine intake, 2014–2021.

Figure 3 shows the geographical distribution of dietary purine intake from 2014 to 2021. It was observed that the average daily purine intake for different regions of China significantly varied, as displayed in a geographical heat map. The highest dietary intake of purine was observed for the population in Southern China, followed by the population in Eastern and Southwestern China. The population in Northwestern China has the lowest dietary intake of purine.

### 3.4. Contribution of different foods consumption to the daily purine intake

**Figure 4:**
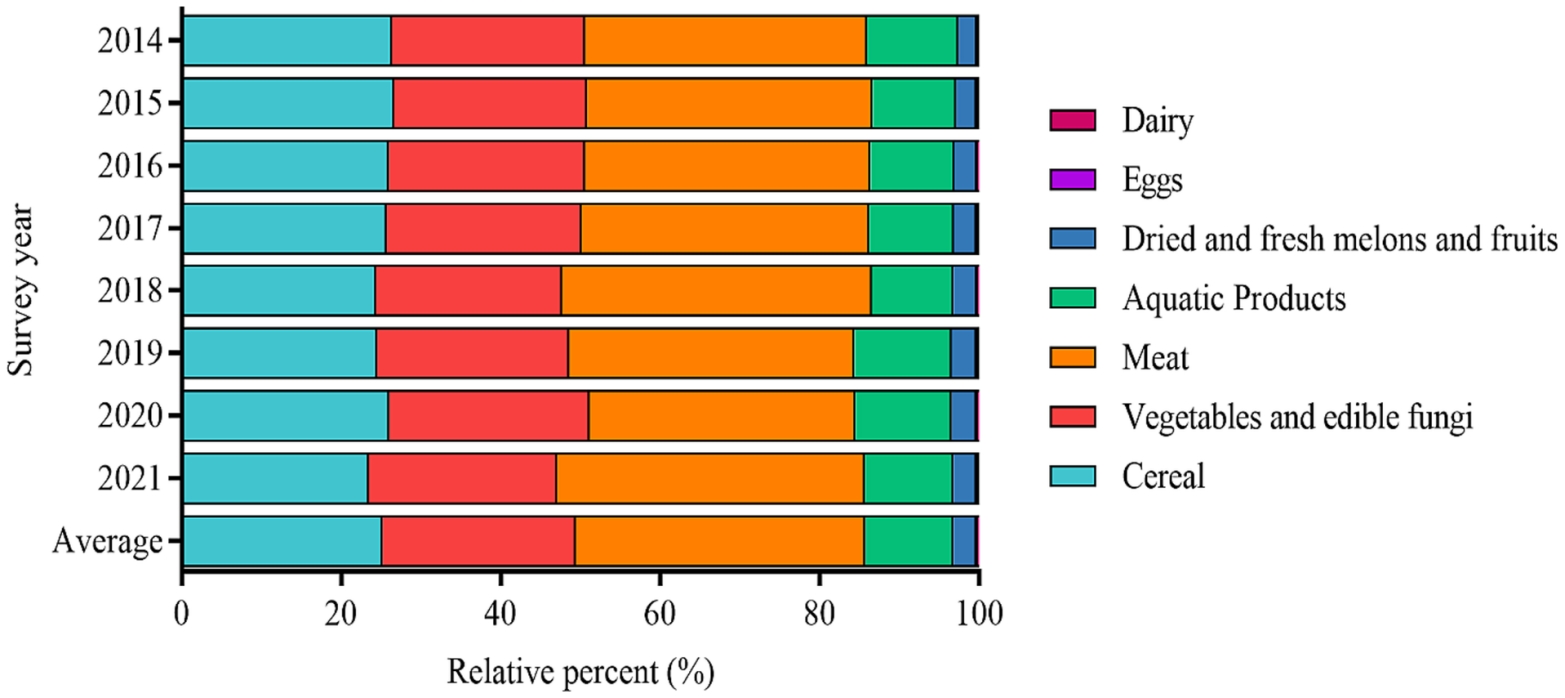
Contribution of different foods consumption to the daily purine intake.

Figure 4 depicts averaged purine intake profiles (2014–2021) in seven food categories. In all the survey years (2014–2021), meat has the largest contribution to the dietary intake of purine (36.2%). Cereals (25.3%) and vegetables and edible fungi (24.2%) consumption have a comparable contribution rate to the dietary intake of purine. Though aquatic products have a high purine content, it has a modest contribution rate (11.0%) to the daily dietary intake of purine for Chinese residents. The above trends were observed in most of the survey year.

## 4. Discussion

Our research found that from 2014 to 2021, the *per capita* total purine intake of Chinese residents showed an overall upward trend. The average daily intake increased from 477.3 mg to 509.2 mg. Dietary meat products showed the highest average contribution rate (32.45%) of dietary purine, followed by vegetables and edible fungi (21.97%) and grains (21.90%).

Between 2014 to 2021, China’s economy developed from high-speed growth to high-quality development, and the *per capita* disposable income of the population showed a yearly growth (23). The eating and consumption habits of Chinese residents have experienced great changes (24). It is manifested that refined grain consumption significantly decreased, whereas coarse grain consumption increased (25). In addition, staple food varieties showed diversification; animal food, fresh fruits, and vegetables consumption has also increased significantly. Compared to 2016, the Pagoda of Balanced Diet for Chinese Residents (2022) subdivided staple foods and separated tubers and cereals, highlighting the importance of tubers and roots. Due to environmental or economic constraints in different regions, animal foods are consolidated so that their inhabitants can more easily implement them to meet nutritional requirements. Simultaneously, the recommended intake of dairy products has also increased. Compared with the data in 2021, the intake of milk, vegetables and edible fungi, dried and fresh melons and fruits, and tubers and roots of Chinese residents did not meet the requirements, while the intake of cereals exceeded recommended values.

At the same time, different storage conditions and processing methods also affect the purine content of foods. Some researchers have studied the effects of photodynamic treatment on the storage life and purine content of *Litopenaeus vannamei* under low-temperature conditions (26). HPLC was performed to determine the shelf-life indices of foods. The results showed that the storage life of shrimp could be extended from 3 to 8 days by microcrystalline refrigeration. Photodynamic treatment combined with microcrystalline storage prolonged the storage life of shrimp to 12 days, and hypoxanthine content decreased significantly after 24 h of storage (26).

Moreover, different processing methods have different effects on the purine content of foods (27). Previous studies have shown the changes in purine content in food by different cooking methods, such as high-temperature heating, boiling, steaming, roasting, ultrasound, and microwaving. Reverse-phase HPLC (RP-HPLC) and ultraviolet detection (UV) were used to analyzes the effects of cooking (including various combinations of boiling, roasting, blanching, baking, and oven drying) on the purine content (including adenine, guanine, xanthine, and hypoxanthine) and their metabolites (uric acid) in three insects (*Tenebrio molitor*, *Gryllus assimilis*, and *Acheta domesticus*) suitable for human consumption (28, 29). According to the data obtained, boiling for 15 min significantly reduced the purine content of *T. molitor* but did not affect the purine content of *A. domesticus* and *G. assimilis*. In contrast, after baking (especially at 220°C), the purine content increased in all insects (30).

Shiitake mushrooms are popular worldwide, and this food is also considered purine-rich. However, the types of purines and changes they undergo during food processing have received little attention. The effects of baking drying, freeze-drying, and sun drying on purine content in Lentinula edodes were compared using acid hydrolysis and HPLC (28). The results showed that adenine content decreased after drying at 120°C, possibly due to thermal damage to the DNA. The total purine content decreased significantly after lyophilisation but remained unchanged after drying and sun exposure. The effects of moisture and heat on the purines of Lentinula edodes were studied. An increase in xanthine content led to an increase in total purine content. The purine content of the cooking liquid was higher than that of the solid. Compared with drying methods, lyophilisation significantly affected purine release and reduced purine content in Lentinula edodes. Therefore, lyophilisation is more suitable for patients with hyperuricemia and gout (28). By understanding the purine content of various foods, consumers can choose foods with low-purine content and reduce their intake in their diets. Food’s low, medium, and high-purine contents are generally less than 50 mg/100 g, 50–150 mg/100 g, and 150–1,000 mg/100 g, respectively.

In interpreting the results of this study, several limitations warrant consideration. Firstly, the dietary evaluation was derived from a predefined set of 10 items within the survey, potentially omitting other relevant dietary contributors to purine intake. Furthermore, this analysis did not account for variations in purine content resulting from different food preparation and cooking methods, both of which can modulate the nutritional profile of food items. Given these constraints, the extrapolation of these findings to a broader dietary context should be approached with caution. Comprehensive assessments encompassing a wider array of food items and considering food preparation nuances are recommended for more detailed elucidation of dietary patterns and their consequent impact on serum uric acid concentrations.

In conclusion, Chinese residents’ total daily purine intake gradually increased from 2014 to 2021. Meat products accounted for the largest proportion of the total dietary intake of purine. Our research underscores the nuanced relationship between China’s socioeconomic progression and its shifting dietary patterns concerning purine intake. Recognizing the potential health implications of increased purine consumption, such as heightened gout risk, we recommend targeted public health campaigns to educate various population segments. This study also illuminates the need for deeper explorations into micro-level factors influencing these dietary shifts, including cultural and regional determinants. While our approach is primarily epidemiological, it’s crucial to note its foundational role in paving the way for multidisciplinary inquiries. By highlighting these trends, we aim to catalyze a broader academic dialog on the implications of China’s evolving dietary landscape.

## Data availability statement

The original contributions presented in the study are included in the article/Supplementary material, further inquiries can be directed to the corresponding authors.

## Author contributions

SL: Formal analysis, Visualization, Writing - original draft. XL: Conceptualization, Methodology, Funding acquisition, Writing - review & editing. XJ: Methodology, Resources, Writing - review & editing. MF: Methodology, Writing - review & editing. QY: Methodology, Supervision, Writing - original draft. ZG: Supervision, Resources, Writing - review & editing.

## Footnotes

^1^www.cbndata.com
