# Supplementary material for: Assessment of the temporal trend and daily profiles of the dietary purine intake among Chinese residents during 2014 to 2021
Source: Front Nutr. 2023 Nov 9;10:1259053. doi: 10.3389/fnut.2023.1259053 (PMC10666749; doi:10.3389/fnut.2023.1259053)
Supplement: Supplementary file 1 [file Data_Sheet_1.docx]

Supplementary Material

**
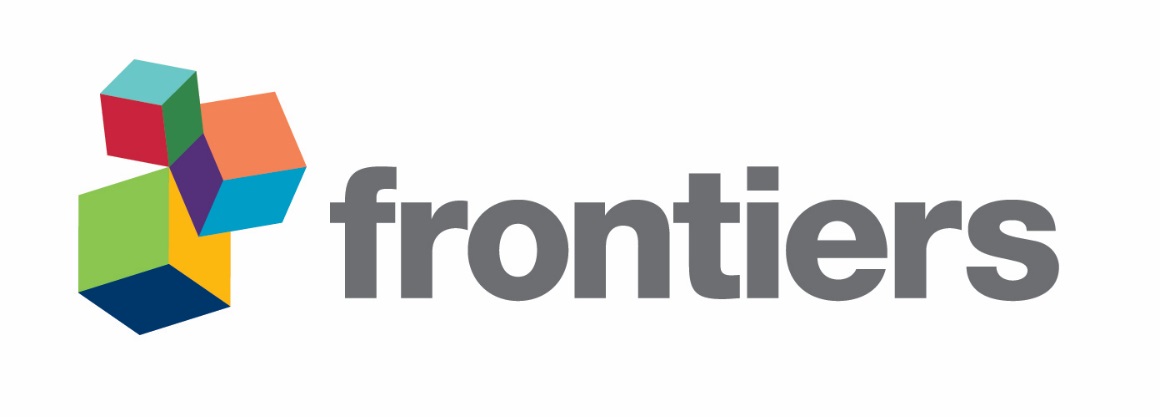
**

**Assessment of the temporal trend and daily profiles of the dietary purine intake among Chinese residents during 2014 to 2021**

Shiwen Li ^1^, Xin Liu ^1*^, JiaXi Wu ^1*^, Min Fang ^1^, Qing Yang^1^

^1^ College of Food Science and Engineering, Key Laboratory for Deep Processing of Major Grain and Oil (The Chinese Ministry of Education), Hubei Key Laboratory for Processing and Transformation of Agricultural Products, Wuhan Polytechnic University, Wuhan, 430023, China;

***Correspondence:**Prof. Xin Liu (E-mail: liuxinhook@126.com; Tel: +086-27-83924790; Fax: +086-83924790). Postal address: No.68 Xuefu South Road, Changqing Garden, Wuhan City, Hubei Province, P.R China, 430023.

Prof. Xiwu Jia (E-mail: jiaxiwu212@126.com; Tel: +086-27-83924790; Fax: +086-83924790). Postal address: No.68 Xuefu South Road, Changqing Garden, Wuhan City, Hubei Province, P.R China, 430023

*, These authors contribute equally to this work.

**Table S1**

| Per Captia Consumption on different foods (Kilogram) | 2021 | 2020 | 2019 | 2018 | 2017 | 2016 | 2015 | 2014 |
| --- | --- | --- | --- | --- | --- | --- | --- | --- |
| **Nationwide** | |  |  |  |  |  |  |  |
| Grain | 144.6 | 141.2 | 130.1 | 127.2 | 130.1 | 132.8 | 134.5 | 141 |
| Edible Oil | 10.8 | 10.4 | 9.5 | 9.6 | 10.4 | 10.6 | 10.6 | 10.4 |
| Vegetable and Edible Mushroom | 109.8 | 103.7 | 98.6 | 96.1 | 99.2 | 100.1 | 97.8 | 96.9 |
| Meat and Products | 32.9 | 24.8 | 26.9 | 29.5 | 26.7 | 26.1 | 26.2 | 25.6 |
| Poultry | 12.3 | 12.7 | 10.8 | 9 | 8.9 | 9.1 | 8.4 | 8 |
| Aquatic Products | 14.2 | 13.9 | 13.6 | 11.4 | 11.5 | 11.4 | 11.2 | 10.8 |
| Eggs | 13.2 | 12.8 | 10.7 | 9.7 | 10 | 9.7 | 9.5 | 8.6 |
| Milk and Diary Products | 14.4 | 13 | 12.5 | 12.2 | 12.1 | 12 | 12.1 | 12.6 |
| Dried and Fresh Melons and Fruits | 61 | 56.3 | 56.4 | 52.1 | 50.1 | 48.3 | 44.5 | 42.2 |
| Sugar | 1.3 | 1.3 | 1.3 | 1.3 | 1.3 | 1.3 | 1.3 | 1.3 |
| **Urban** |  |  |  |  |  |  |  |  |
| Grain | 124.8 | 120.2 | 110.6 | 110 | 109.7 | 111.9 | 112.6 | 117.2 |
| Edible Oil | 10.1 | 9.9 | 9.2 | 9.4 | 10.7 | 11 | 11.1 | 11 |
| Vegetable and Edible Mushroom | 112 | 109.8 | 105.8 | 103.1 | 106.7 | 107.5 | 104.4 | 104 |
| Meat and Products | 34.4 | 27.4 | 28.7 | 31.2 | 29.2 | 29 | 28.9 | 28.4 |
| Poultry | 12.3 | 13 | 11.4 | 9.8 | 9.7 | 10.2 | 9.4 | 9.1 |
| Aquatic Products | 16.7 | 16.6 | 16.7 | 14.3 | 14.8 | 14.8 | 14.7 | 14.4 |
| Eggs | 13.4 | 13.5 | 11.5 | 10.8 | 10.9 | 10.7 | 10.5 | 9.8 |
| Milk and Diary Products | 18.2 | 17.3 | 16.7 | 16.5 | 16.5 | 16.5 | 17.1 | 18.1 |
| Dried and Fresh Melons and Fruits | 67.7 | 65.9 | 66.8 | 62 | 59.9 | 58.1 | 55.1 | 52.9 |
| Sugar | 1.1 | 1.2 | 1.2 | 1.3 | 1.3 | 1.3 | 1.3 | 1.3 |
| **Rural** |  |  |  |  |  |  |  |  |
| Grain | 170.8 | 168.4 | 154.8 | 148.5 | 154.6 | 157.2 | 159.5 | 167.6 |
| Edible Oil | 11.7 | 11 | 9.8 | 9.9 | 10.1 | 10.2 | 10.1 | 9.8 |
| Vegetable and Edible Mushroom | 107 | 95.8 | 89.5 | 87.5 | 90.2 | 91.5 | 90.3 | 88.9 |
| Meat and Products | 30.9 | 21.4 | 24.7 | 27.5 | 23.6 | 22.7 | 23.1 | 22.5 |
| Poultry | 12.4 | 12.4 | 10 | 8 | 7.9 | 7.9 | 7.1 | 6.7 |
| Aquatic Products | 10.9 | 10.3 | 9.6 | 7.8 | 7.4 | 7.5 | 7.2 | 6.8 |
| Eggs | 13 | 11.8 | 9.6 | 8.4 | 8.9 | 8.5 | 8.3 | 7.2 |
| Milk and Diary Products | 9.3 | 7.4 | 7.3 | 6.9 | 6.9 | 6.6 | 6.3 | 6.4 |
| Dried and Fresh Melons and Fruits | 52.4 | 43.8 | 43.3 | 39.9 | 38.4 | 36.8 | 32.3 | 30.3 |
| Sugar | 1.5 | 1.4 | 1.4 | 1.3 | 1.4 | 1.4 | 1.3 | 1.3 |

**Table S2. The calculated average purine content in different types of food (mg/100g)**

| Food Category | Purine content (mg/100g) | | | |
| --- | --- | --- | --- | --- |
|  | Mean | Standard Deviation | 25th Percentile | 75th Percentile |
| Cereals | 31.90 | 17.52 | 16.25 | 41.75 |
| Tubers | 14.40 | 4.92 | 12.00 | 13.00 |
| Beans | 200.40 | 62.21 | 156.00 | 218.00 |
| Vegetables and edible fungi | 39.75 | 78.78 | 11.00 | 39.75 |
| Meat | 218.79 | 106.10 | 124.00 | 306.00 |
| Aquatic products | 169.03 | 53.22 | 128.50 | 207.00 |
| Dried and fresh melons and fruits | 8.79 | 2.60 | 1.00 | 2.50 |
| Eggs | 2.50 | 1.22 | 1.00 | 2.50 |
| Dairy | 2.00 | 4.62 | 5.25 | 11.00 |

**Table S3. The purine content in different individual food used to calculate the average level.**

| Category | Foods | Purine content (mg/100g) |
| --- | --- | --- |
| Cereals | Rice | 44 |
|  | Wheat | 22 |
|  | Maize | 12 |
|  | Millet | 20 |
|  | Black Rice | 63 |
|  | Buckwheat | 34 |
|  | Oats | 59 |
|  | Myotonin | 15 |
|  | Sorghum Rice | 15 |
|  | Brown Rice | 35 |
| Tubers | Sweet Potato (Purple Heart) | 24 |
|  | Groundnut | 13 |
|  | Potatoes | 13 |
|  | Sweet Potato | 12 |
|  | Cassava | 10 |
| Beans | Soya Beans | 218 |
|  | Mung Beans | 196 |
|  | Red Beans | 156 |
|  | Fava Beans | 307 |
|  | White Kidney Beans | 125 |
| Vegetables and edible fungi | Carrots | 17 |
|  | White Carrot | 11 |
|  | Cowpea Corner | 45 |
|  | Mung Bean Sprouts | 11 |
|  | Aubergine | 13 |
|  | Cucumber | 11 |
|  | Green Pepper | 6 |
|  | Lettuce | 16 |
|  | Flowering Cabbage | 17 |
|  | Empanada | 22 |
|  | Chinese Cabbage | 14 |
|  | Nectarine | 13 |
|  | Celtuce | 12 |
|  | Spinach | 8 |
|  | Celery | 5 |
|  | Chinese Kale | 19 |
|  | Lotus Root | 10 |
|  | Taro | 15 |
|  | Shiitake Mushrooms | 405 |
|  | Mullein | 38 |
|  | Golden Needle Mushroom | 59 |
|  | Agrocybe Cylindracea | 48 |
|  | Portobello Mushroom | 50 |
|  | Oyster Mushroom | 89 |
| Meat | Chitterlings | 296 |
|  | Pork Liver | 275 |
|  | Pork Belly | 252 |
|  | Pork | 138 |
|  | Pork Trotter | 134 |
|  | Pork Blood | 40 |
|  | Beef | 105 |
|  | Lamb | 109 |
|  | Lamb Kebabs | 223 |
|  | Bullfrog Leg Meat | 92 |
|  | Pork Ears | 114 |
|  | Chicken Liver | 317 |
|  | Chicken Gizzards | 218 |
|  | Chicken | 208 |
|  | Chicken Heart | 168 |
|  | Duck Liver | 398 |
|  | Duck Sausage | 346 |
|  | Duck Gizzard | 316 |
|  | Foie Gras | 408 |
| Aquatic products | Snakehead | 214 |
|  | Salmon | 168 |
|  | Yellow Croaker | 165 |
|  | Crucian | 154 |
|  | Silver Carp | 141 |
|  | Trachinotus Ovatus | 130 |
|  | Eel | 127 |
|  | Mandarin Fish | 121 |
|  | Sardine | 82 |
|  | Soft-shelled Turtle | 110 |
|  | Prawn | 101.5 |
|  | Mantis Shrimp | 200 |
|  | Metapenaeus Ensis | 174 |
|  | Cray | 174 |
|  | River Crab | 147 |
|  | Oyster | 282 |
|  | Scallop | 235 |
|  | Ostracean | 242 |
|  | Squid | 244 |
| Dried and fresh melons and fruits | Bergamot Pear | 5 |
|  | Apple | 1 |
|  | Peach | 14 |
|  | Cherry | 11 |
|  | Plum | 5 |
|  | Grape | 9 |
|  | Tangerine | 9 |
|  | Sugar Orange | 5 |
|  | Litchi | 20 |
|  | Pitaya | 13 |
|  | Pineapple | 11 |
|  | Longan | 7 |
|  | Banana | 7 |
|  | Watermelon | 6 |
| Eggs | Henapple | 1 |
|  | Preserved Duck Egg | 1 |
|  | Goose Egg | 1 |
| Dairy | Milk | 1 |
|  | Yogurt | 4 |
|  | Cheese | 1 |
|  | Milk Powder | 2 |
